# An Outstandingly Rare Occurrence of Mycoviruses in Soil Strains of the Plant-Beneficial Fungi from the Genus *Trichoderma* and a Novel *Polymycoviridae* Isolate

**DOI:** 10.1128/spectrum.05228-22

**Published:** 2023-04-06

**Authors:** Chenchen Liu, Xiliang Jiang, Zhaoyan Tan, Rongqun Wang, Qiaoxia Shang, Hongrui Li, Shujin Xu, Miguel A. Aranda, Beilei Wu

**Affiliations:** a State Key Laboratory for Biology of Plant Diseases and Insect Pests, Institute of Plant Protection, Chinese Academy of Agricultural Sciences, Beijing, China; b Key Laboratory for Northern Urban Agriculture of Ministry of Agriculture and Rural Affairs, Beijing University of Agriculture, Beijing, China; c College of Horticulture and Landscapes, Tianjin Agricultural University, Tianjin, China; d Department of Stress Biology and Plant Pathology, Centro de Edafología y Biología Aplicada del Segura (CEBAS)-CSIC, Murcia, Spain; University of Natural Resources and Life Sciences Vienna

**Keywords:** biocontrol, genome sequence, polymycovirus, protein structure, *Trichoderma barbatum*, TbPMV1, *Trichoderma* spp.

## Abstract

In fungi, viral infections frequently remain cryptic causing little or no phenotypic changes. It can indicate either a long history of coevolution or a strong immune system of the host. Some fungi are outstandingly ubiquitous and can be recovered from a great diversity of habitats. However, the role of viral infection in the emergence of environmental opportunistic species is not known. The genus of filamentous and mycoparasitic fungi *Trichoderma* (Hypocreales, Ascomycota) consists of more than 400 species, which mainly occur on dead wood, other fungi, or as endo- and epiphytes. However, some species are environmental opportunists because they are cosmopolitan, can establish in a diversity of habitats, and can also become pests on mushroom farms and infect immunocompromised humans. In this study, we investigated the library of 163 *Trichoderma* strains isolated from grassland soils in Inner Mongolia, China, and found only four strains with signs of the mycoviral nucleic acids, including a strain of *T. barbatum* infected with a novel strain of the *Polymycoviridae* and named and characterized here as Trichoderma barbatum polymycovirus 1 (TbPMV1). Phylogenetic analysis suggested that TbPMV1 was evolutionarily distinct from the *Polymycoviridae* isolated either from Eurotialean fungi or from the order *Magnaportales*. Although the *Polymycoviridae* viruses were also known from Hypocrealean Beauveria bassiana, the phylogeny of TbPMV1 did not reflect the phylogeny of the host. Our analysis lays the groundwork for further in-depth characterization of TbPMV1 and the role of mycoviruses in the emergence of environmental opportunism in *Trichoderma*.

**IMPORTANCE** Although viruses infect all organisms, our knowledge of some groups of eukaryotes remains limited. For instance, the diversity of viruses infecting fungi—mycoviruses—is largely unknown. However, the knowledge of viruses associated with industrially relevant and plant-beneficial fungi, such as *Trichoderma* spp. (Hypocreales, Ascomycota), may shed light on the stability of their phenotypes and the expression of beneficial traits. In this study, we screened the library of soilborne *Trichoderma* strains because these isolates may be developed into bioeffectors for plant protection and sustainable agriculture. Notably, the diversity of endophytic viruses in soil *Trichoderma* was outstandingly low. Only 2% of 163 strains contained traces of dsRNA viruses, including the new Trichoderma barbatum polymycovirus 1 (TbPMV1) characterized in this study. TbPMV1 is the first mycovirus found in *Trichoderma*. Our results indicate that the limited data prevent the in-depth study of the evolutionary relationship between soilborne fungi and is worth further investigation.

## INTRODUCTION

*Trichoderma* spp. (Hypocreales, Ascomycota) are mycoparasitic filamentous fungi that occur on other fungi, dead wood, and in soils. Some of them inhabit a diverse range of ecological niches in plants as endo- and epiphytes and thus are considered environmental opportunists (1). *Trichoderma* spp. are commonly used as agents of biological control of fungal pests (biocontrol) (2–5) in sustainable agriculture because of their ability to parasitize plant-pathogenic fungi. They are especially effective in controlling soilborne diseases, such as root rot caused by *Rhizoctonia* spp., *Verticillium* spp., and Fusarium spp. (6–9). There is also evidence to suggest that *Trichoderma* species can promote plant growth and induce systemic resistance (10–14). The comparative genomic studies of several opportunistic strains of *Trichoderma* revealed numerous properties, which can be associated with their ecological versatility but did not explain them (1, 15, 16). In this study, we tested whether the soil strains of *Trichoderma* contain viruses as our previous studies showed that this may influence their phenotype (12, 14).

Viruses that infect and replicate in filamentous fungi and yeasts (mycoviruses) are found among all major groups of fungi and fungi-like protozoans (17, 18). Despite the high application value of *Trichoderma* spp. in biocontrol and plant growth promotion, mycoviruses have only been studied in a few randomly selected isolates. Currently, only 11 isolates of mycoviruses have been explored from *Trichoderma* spp., which are all dsRNA nucleotide forms from *Partitiviridae*, *Hypoviridea*, *Fusagraviridae*, and other yet undefined families (12, 14, 19–27) (Table 1).

**TABLE 1:** Mycoviruses from *Trichoderma* spp.

Name	Abbreviation	Taxonomy	*Trichoderma* host	Reference
Trichoderma atroviride mycovirus 1	TaMV1	Unclassified	T. atroviride	19
Trichoderma atroviride partitivirus 1	TaPV1	*Partitiviridae*	T. atroviride	20
Trichoderma harzianum partitivirus 1	ThPV1	*Partitiviridae*	*T. harzianum*	21
Trichoderma asperellum dsRNA Virus 1	TaRV1	Unclassified	*T. asperellum*	22
Trichoderma koningiopsis totivirus 1	TkTV1/Mg10	*Hypoviridea*	*Trichoderma koningiopsis*	23
Trichoderma harzianum hypovirus 1	ThHV1	*Hypoviridae*	*Trichoderma harzianum*	24
Trichoderma harzianum bipartite mycovirus 1	ThBMV1	Unclassified	*Trichoderma harzianum*	25
Trichoderma harzianum mycovirus 1	ThMV1	Unclassified	*Trichoderma harzianum*	19
Trichoderma atroviride mycovirus 1-NFCF377	TaMV1-NFCF377	*Fusagraviridae*	*Trichoderma atroviride*	20
Trichoderma harzianum hypovirus 2	ThHV2	*Hypoviridae*	*Trichoderma harzianum*	21
Trichoderma harzianum partitivirus 2	ThPV2	*Partitiviridae*	*Trichoderma harzianum*	22

Most mycoviruses have no significant influence on the colony morphology and mycelial growth rate of *Trichoderma* spp. (20, 21, 25, 26), except for the research of Wang et al. on Trichoderma harzianum partitivirus 2, or ThPV2 (14), which caused faster growth of the host strain and triggered abundant aerial hyphae and dark green pigmentation of the conidia. However, the infection of *T. harzianum* with ThPV2 did not change the efficiency of the biocontrol product based on this fungus. In other reports, viruses affected the secondary metabolism of *Trichoderma* (26) or influenced the activity of lignocellulolytic enzymes (20). Some mycoviruses, such as ThPV2 (14), altered the biomass and spore production by *Trichoderma* spp. (14, 25, 28, 29); similar to the effects of Fusarium graminearum virus-china 9 (FgV-ch9) (28) and Rhizoctonia solani dsRNA virus 5 (RsRV5) (29) on their hosts.

Fusarium spp. (Hypocreales, Ascomycota) and Sclerotinia sclerotiorum (Helotiales, Ascomycota) are mainly soilborne plant-pathogenic fungi (30, 31). To date, mycoviruses with whole-genome sequences have been reported in 13 Fusarium species, including Fusarium graminearum virus 1 (FgV1), FgV-ch9, Fusarium graminearum hypovirus 2 (FgHV2), and Fusarium oxysporum f. sp. dianthi mycovirus 1 (FodV1), which have been associated with hypovirulence in plants (31–34). In the population of mycoviruses with 68 isolates from *S. sclerotiorum* in the three successive years checked, 24 isolates were detected repeatedly, and two-thirds of the mycoviruses had positive-sense, single-stranded RNA genomes (35).

Soil-borne *Trichoderma* spp. may be putatively suitable bioeffectors for agriculture (36). To study the diversity of mycoviruses, we tested 163 soil isolates. Notably, only four strains contained mycoviruses, including the new virus from *T. barbatum*. The genome and predicted protein products of this mycovirus were characterized and the relationships with other mycoviruses were analyzed.

## RESULTS

### One strain of *Trichoderma* out of 160 was infected by a virus.

**FIG 1:**
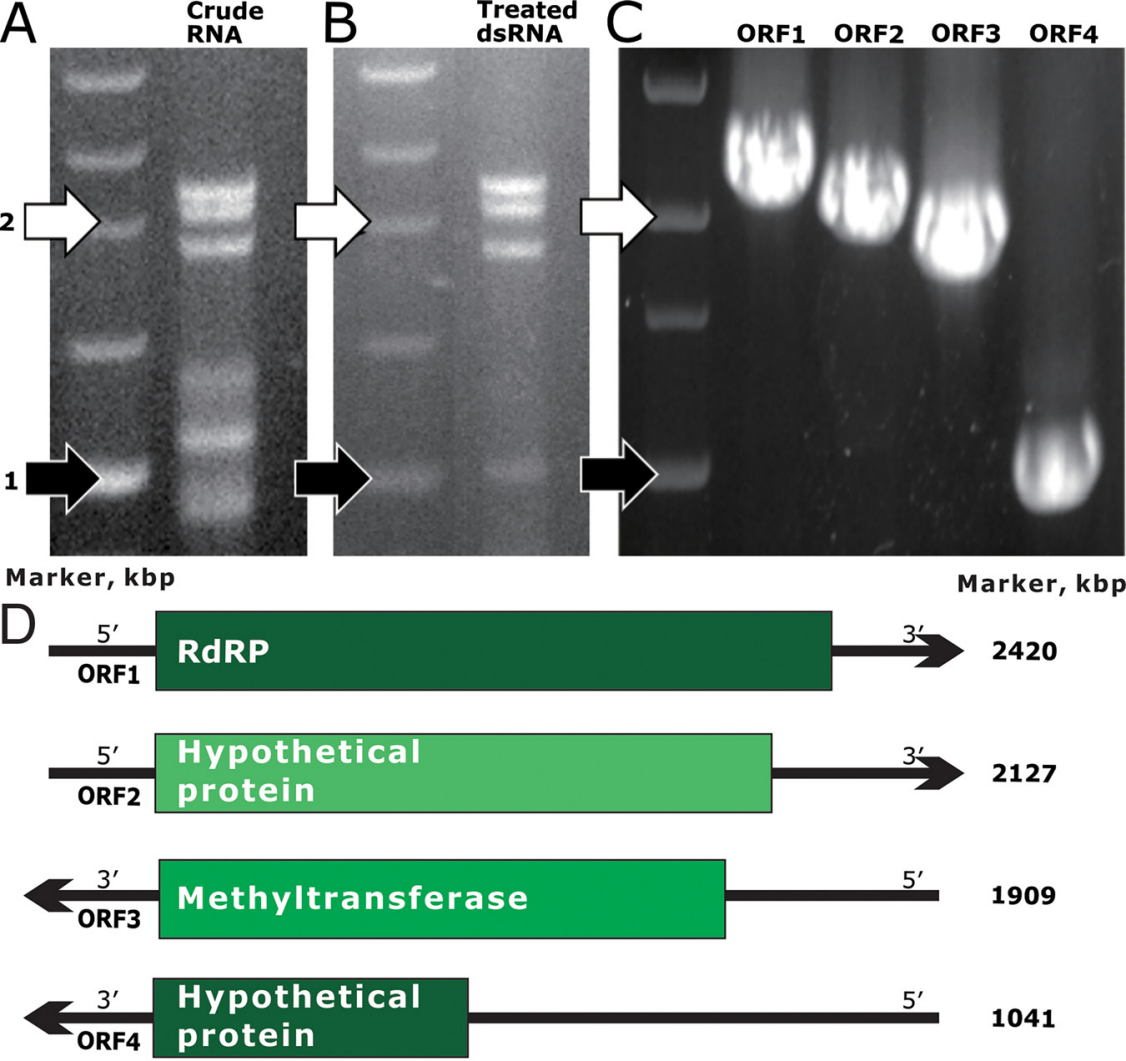
Isolation of the dsRNA virus from *Trichoderma barbatum* HB40111 and the structure of its genome. (A) Untreated dsRNA; (B) DNase I- and S1-treated dsRNA; (C) viral genome fragments detected via RT-PCR; (D) the schematic representation of the genome organization of Trichoderma barbatum polymycovirus 1 (TbPMV1).

One hundred sixty *Trichoderma* species isolates were tested for the presence of RNA viruses, and only a single strain—HB40111—shows a positive result (Fig. 1A and B). The three-loci DNA barcoding analysis as specified by Cai and Druzhinina (37) identified the strain HB40111 of *T. barbatum* as *tef1* (NCBI GenBank accession number OP978144) and *rpb2* (NCBI GenBank accession number OP978145) with sequences that were > 97% and 99% similar to the sequences of the ex-type strain of this species (CBS 125733), respectively; ITS (NCBI GenBank accession number OP978249) was also identical to the sequence of CBS 125733. Diversity analysis using ITS rRNA sequences revealed that there are no other strains with identical or highly similar phylotypes, meaning that only one isolate of *T. barbatum* was sampled (Table S1 and Fig. S1). The detailed molecular identification of the 159 virus-free strains is presented elsewhere. In our previous study, we detected mycoviruses in the three other strains from the same data set, including two distinct strains of *T. harzianum* infected with Trichoderma harzianum bipartite mycovirus 1 (25) and Trichoderma harzianum mycovirus 1 (12). Furthermore, *Trichoderma* sp. HB40444 contained a putative dsRNA mycovirus, which could not be identified (data not shown). Thus, the 163 *Trichoderma* strains isolated from grassland soils in Inner Mongolia contained a maximum of four isolates with putative viral infections.

### *T. barbatum* HB40111 was infected with the dsRNA virus.

The metagenomic sequencing and the electrophoretic analysis revealed that the sizes of the mycovirus fragments are approximately 2.4 kb, 2.1 kb, 1.9 kb, and 1 kb (Fig. 1). We screened dsRNA contigs with a similar band size and homology to fungal mycoviruses. Based on the sizes of the fragments (2.4 kb, 2.1 kb, 1.9 kb, and 1 kb), four virus contigs with similar sizes were screened and searched for similar isolates from the BLASTN of NCBI (https://blast.ncbi.nlm.nih.gov/Blast.cgi?PROGRAM=tblastn&PAGE_TYPE=BlastSearch&LINK_LOC=blasthome), and three fungal mycoviruses had homology with it: Aspergillus fumigatus tetramycovirus-1, Cladosporium cladosporioides virus 1, and Botryosphaeria dothidea virus 1. RT-PCR analysis verified that the extracted dsRNA contains the screened contigs (Fig. 1). The sequencing results were consistent with the metagenomics sequencing results. The 5′ end sequence and 3′ end sequence of the cDNA were obtained using the classical 5′ RACE and 3′ RACE cloning methods, and the 3′ end of the virus contained a poly(A) structure for every fragment. The complete genome sequencing of the virus was assembled: dsRNA1 was 2420 bp, dsRNA2 was 2127 bp, dsRNA3 was 1909 bp, and dsRNA4 was 1041 bp. The GC content of each segment is 56.7%, 58.3%, 55.7%, and 61.5%. The NCBI ORF Finder predicted that the dsRNA1 and dsRNA2 coding strands contain one ORF each on the positive strand, whereas dsRNA3 and dsRNA4 have one ORF each on the negative strand. ORF1 (nt positions 33 to 2333) was predicted to encode a protein of 766 amino acids (aa) in length and ~84.7 kDa with RNA-dependent RNA polymerase (RdRP) activity. ORF2 (nt positions 75 to 2051) was predicted to encode a hypothetical protein of 678 aa in length and ~71.1 kDa, and ORF3 (nt 1857 to 16) was predicted to encode a protein of 613 aa in length and ~65.5 kDa with methyl transferase activity. Finally, ORF4 (nt positions 932 to 150) was predicted to encode a hypothetical protein of 260 aa in length and ~27.7 kDa. These nucleotide sequences and amino acid sequences have been submitted to GenBank under the accession numbers OM307406, OM307407, OM307408, and OM307409. The 5′ UTRs of dsRNA1, dsRNA2, dsRNA3, and dsRNA4 are 32, 74, 52, and 109 bp long, respectively (Fig. S2, S4, S6, and S8); the 3′UTRs are 87, 76, 15, and 149 bp (Fig. 1; Fig. S3, S5, S7, S9, and S8).

### The mycovirus isolated from *T. barbatum* HB40111belonged to the family *Polymycoviridae*.

**FIG 2:**
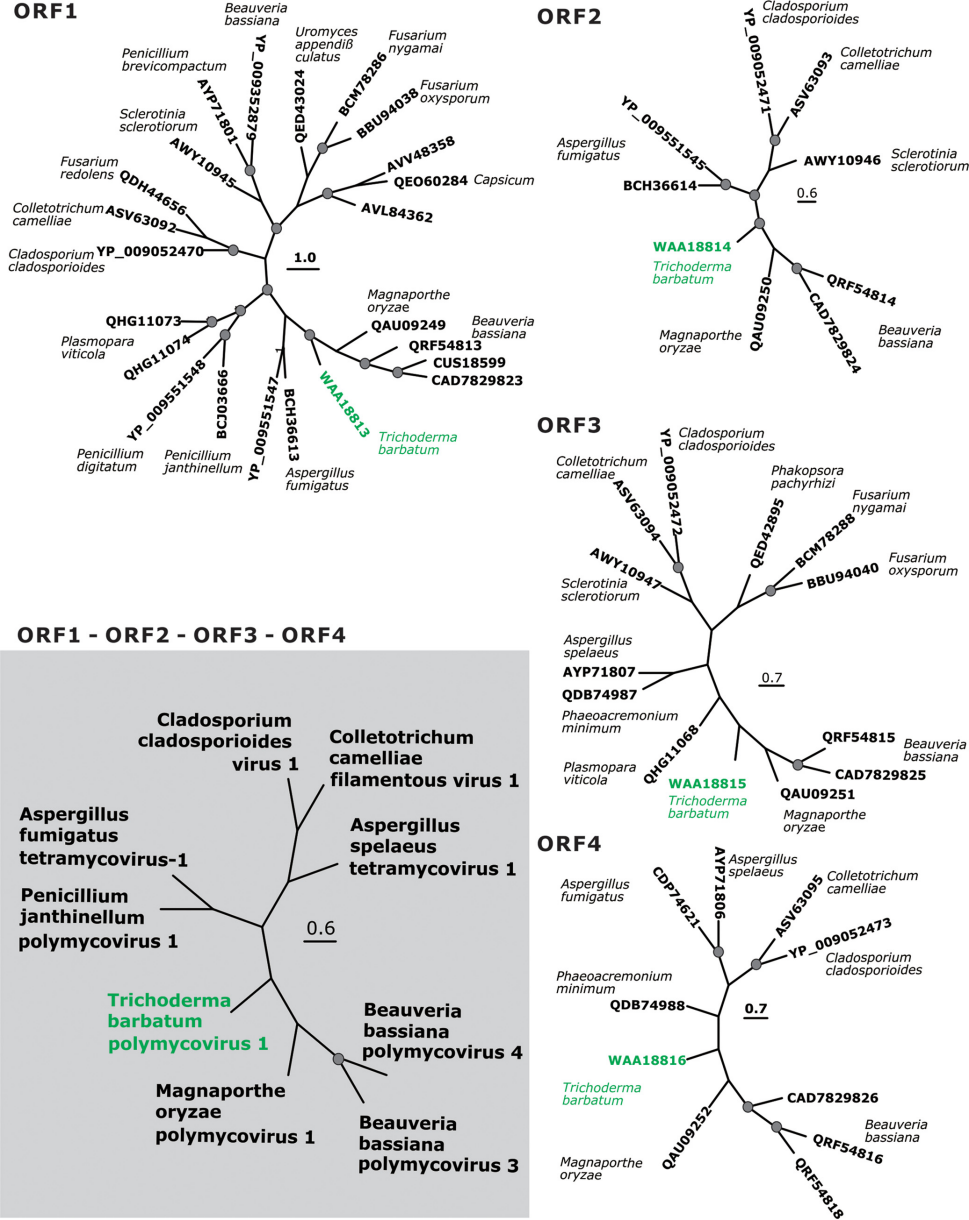
Phylogenetic trees based on the amino acid sequences of proteins encoded by ORF1, ORF2, ORF3, ORF4. NCBI GenBank accession numbers for OTUs of respective viruses are supplemented with the Latin names of the host fungi given in italics. The phylogenetic position of TbPMV1 is presented on the shaded phylogram based on the concatenated alignment of all four sequences. Nodes with significant bootstrap support are covered by the gray dot (>60%).

BLAST was used to identify homologs of ORF 1. The four most closely related mycovirus proteins of RdRPs were encoded by Magnaporthe oryzae polymycovirus 1 (MoPmV-1) (QAU09249.1; 52.88% similarity), Beauveria bassiana polymycovirus 2 (BbPmV-2) (CUS18599.1; 54.85% similarity), Beauveria bassiana polymycovirus 3 (BbPmV-3) (38) (CAD7829823.1; 55.50% similarity), and Beauveria bassiana polymycovirus 4 (BbPmV-4) (39) (QRF54813.1; 55.74% similarity). Therefore, ORF 1 was speculated as RdRP (accession number from NCBI: WAA18813). A phylogenetic tree was constructed based on the RdRP sequences of these five closely related mycoviruses and 18 other mycoviruses from different groups (*Polymycoviridae* and unclassified viruses). As expected, MoPmV-1, BbPmV-2, BbPmV-3, BbPmV-4, and the mycovirus isolated from *T. barbatum* strain HB40111formed one *Polymycoviridae* clade and were genetically separated from the other mycoviruses (Fig. 2 and Table S3). Finally, the I-TASSER analysis indicates that the likely ligand-binding sites of RdRP are P332 and K333 (C-score = 0.04) and the probable active site residue is E670 (Cscore^EC^ = 0.111) (Table 2 and Fig. S10).

**TABLE 2:** I-TASSER analysis for the likely ligand-binding sites and the likely ligand-binding sites of the proteins of TbPMV1

Protein	Likely ligand-binding sites	Probable active site residue with parameter
ORF 1	P332 and K333	E670 (CscoreEC = 0.111)
RNA-dependent RNA polymerase	(CscoreEC = 0.111)	
ORF 2	K34, T82, and A85	No active site residues
Transmembrane transporter activity	(C-score = 0.06)	
ORF 3	L163 and L166	No active site residues
Hypoprotein	(C-score = 0.09)	
ORF 4	S22, V23, A24, L128, G130, E168, A169, and V17	No active site residues
Hypoprotein	(C-score = 0.10)	

The three mycovirus proteins are most similar to the hypothetical protein encoded by BbPmV-2 (QRF54814.1; 50.73% similarity), BbPmV-3 (CAD7829824.1; 49.71% similarity), and MoPmV-1 (QAU09250.1; 49.27% similarity), and phylogenetic analysis of five other mycoviruses (*Polymycoviridae* and unclassified viruses) was conducted. The resulting ML tree shows that the mycovirus isolated from *T. barbatum* strain HB40111 is clustered together with *Polymycoviridae* (Fig. 2 and Table S4). I-TASSER analysis indicates that the protein encoded by ORF2 (WAA18814) has transmembrane transporter activity; the putative ligand-binding site residues are K34, T82, and A85 (C-score = 0.06); and there are no active site residues (Table 2 and Fig. S11).

For ORF3 (WAA18815), the four most closely related mycovirus proteins are present in BbPmV-4 (QRF54815.1; 45.67% similarity), BbPmV-3 (CAD7829825.1; 47.03% similarity), MoPmV-1 (CAD7829825.1; 41.58% similarity), and Phaeoacremonium minimum tetramycovirus 1 (PmTMV1) (40) (QDB74987.1; 43.25% identity). A phylogenetic tree was constructed with four closely related mycoviruses and homologous sequences from the other 10 mycoviruses from different groups (*Polymycoviridae* and unclassified viruses). Based on the best substitution model (WAG+G) (Table S2), the mycovirus from *T. barbatum* HB40111 clustered with *Polymocoviridae* (Fig. 4C and Table S5). I-TASSER predicted that the ligand-binding site residues are L163 and L166 (C-score = 0.09) with no active site residues (Table 2 and Fig. S12).

The mycovirus species encoding the four most closely related proteins to the predicted product of ORF4 (WAA18816) are the same as those for ORF3: BbPmV-4 (QRF54816.1; 49.03% similarity), BbPmV-3 (CAD7829826.1; 47.67% similarity), MoPmV-1 (QAU09252.1; 41.53% similarity), and PmTMV1 (QDB74988.1; 45.04% similarity). The best substitution model was LG+G+F (Table S2). As with the other three ORFs, the mycovirus from *T. barbatum* BH40111 clustered with *Polymycoviridae* mycoviruses (Fig. 2). I-TASSER predicted that the ligand-binding site residues are S22, V23, A24, L128, G130, E168, A169, and V17 (C-score = 0.10) with no active site residues (Table 2 and Fig. S13).

Finally, we constructed a phylogenetic tree based on the concatenated ORF1, ORF2, ORF3, and ORF4 alignment (Fig. 2). The resulting tree topology was consistent with those of the single ORF-based trees. Based on these results, we conclude that the novel mycovirus from *T. barbatum* BH40111 is a polymycovirus, which is the first mycovirus of *T. barbatum*; therefore, we propose the name Trichoderma barbatum polymycovirus 1 (TbPMV1). However, TbPMV1 has a distinct phylogenetic position surrounded by viruses isolated from fungi from another class (Eurotiomycetes) or Magnaportales order of Sordariomycetes. The viruses from the phylogenetically close *Trichoderma* hosts belonged to the same subclade of the *Polymycoviridae* but are genetically distinct (Fig. 2).

## DISCUSSION

Members of the *Polymycoviridae* family are unique and possess multisegmented dsRNA genomes (from 3 to 11 segments) (34, 41–43) and noncapsid proteins (44). In this study, we discovered a novel mycovirus species, TbPMV1, a *Polymycoviridae* member present in the *T. barbatum* strain isolated from the soil. This is the first polymycovirus identified in the genus *Trichoderma*, enriching the known diversity of mycoviruses associated with these fungi (Table 1). Before our study, 11 *Trichoderma* mycoviruses have been reported, which have shown diversity in population, comprising one *Fusagraviridae* (TaMV1-NFCF377), two *Hypoviridae* (ThHV1 and ThHV2), three *Partitiviridae* (ThPV1, TaPV1, and TaPV2), one totivirus (TkTV1/Mg10), and four unclassified (TaMV1, TaRV1, ThBMV1, and ThMV1). Though phylogenetic analysis was carried out for the 12 mycoviruses, the best alignment of each possible protein could not be obtained, which means it is difficult to demonstrate the evolutionary relationship using the limited isolates from *Trichoderma* spp.; we need to explore more mycovirus isolates from this soilborne fungus. Moreover, the discovery of TbPMV1 also provides evidence for the classification and diversity of mycoviruses associated with *Trichoderma* spp.

Because the previous characterizations of *Trichoderma* mycoviruses are limited, there is a dearth of experimentally validated functional data from which the functions of proteins encoded in the genome of TbPMV1 could be inferred. ORF1 has a high similarity to the RdRP of the other *Polymycoviridae* members, so the function of ORF1 should be similar to the function of RdRP. The protein product of ORF2 was annotated as having putative transmembrane transporter activity, which should have the movement function of mycoviruses. There are no predicted functions for proteins encoded by ORF3 and ORF4.

In addition to the characterization of the novel *Polymycoviridae* virus and the first detection of these viruses in *Trichoderma* spp., the most interesting result of this study is the outstandingly rare occurrence of viruses in these fungi. The phylogenetic analysis suggests that these viruses are unlikely to be specific to *T. barbatum* or that the data are not enough to speculate on the possible coevolution between the virus and the host. This is the fourth mycovirus to be isolated from a collection of 163 *Trichoderma* strains in our laboratory, making the positive rate 2.45%. This demonstrates that associations between mycoviruses and *Trichoderma* strains are outstandingly rare. Further screening should reveal whether strictly mycoparasitic strains of *Trichoderma* are also largely virus-free and whether the same pattern can be reproduced for soil *Trichoderma* from another geographic region.

## MATERIALS AND METHODS

### Isolation and molecular identification of *Trichoderma* spp.

One hundred and sixty-three *Trichoderma* species isolates were obtained from grassland soils in Xinjiang and Inner Mongolia provinces from 2014 to 2016 (Table S1). Pure single-spored cultures were stored on potato dextrose agar at 4°C. The diversity of the strains was assessed using the primary fungal DNA barcoding locus (ITS of the rRNA gene cluster) (37, 45), while the precise and accurate molecular identification of these strains is presented elsewhere. The strain containing the new mycovirus was identified using the combination of the ITS and the two secondary DNA barcoding markers (partial fragments encoding the translation elongation factor 1-alpha *tef1* and RNA-polymerase subunit B2 *rpb2*) using the protocols of Cai and Druzhinina (46).

### Extraction and purification of dsRNA.

The diversity of mycoviruses with RNA genomes was tested in the strains that were cultivated in the liquid potato glucose broth at 28°C with 200 rpm shaking for 2 days. Mycelia were collected from the liquid cultures via vacuum filtration (using a 0.22-μm pore size). A standard phenol-chloroform extraction method (47) was used to isolate the RNA, which was then purified using CF11 cellulose column chromatography as previously described (48). The isolated RNA was treated with RNase-free DNase I (TaKaRa, Dalian, China) and S1 Nuclease (TaKaRa) according to the manufacturer’s instructions to remove DNA and single-stranded nucleic acids, respectively. Finally, the dsRNA was visualized on a 1% agarose gel. Moreover, the dsRNA was also degraded by DNase and RNase III; DNase can degrade dsDNA and ssDNA while RNase III can only degrade ssRNA. Therefore, after the degradation, the nucleotide type should be checked out. After the application of DNase, if the fragments disappear the mycovirus should be the ssDNA or dsDNA nucleotide style; if the fragments are retained, the mycovirus should be the dsRNA nucleotide style. After the degradation by RNase III, if the fragments disappear, the mycovirus should be the ssRNA nucleotide style; if the fragments are retained, the mycovirus should be the dsRNA, ssDNA, or dsDNA nucleotide style. Therefore, the nucleotide style of the mycovirus needs to be screened after the degradation by DNase and RNase III.

### DsRNA sequencing.

Before sequencing, dsRNA was further purified using an RNAClean XP kit (Beckman Coulter, Inc., Brea, CA, USA) and a Ribo-Zero rRNA Removal kit (Epicentre, Madison, WI, USA). For each sample, >1 μg of dsRNA was prepared for metagenomic sequencing at the Shanghai Biotechnology Corporation (Shanghai, China) using the Illumina HiSeq 2000 and NovaSeq 6000 platforms (Agilent Technologies, Santa Clara, CA, USA). The sequencing data were then analyzed, which entailed data preprocessing, sequence splicing (49–51), database annotation (www.mg-rast.org), gene quantification, species classification, and species abundance analysis.

### Characterization of the mycovirus genome.

Unigenes were annotated using RefSeq, and proteins were annotated using the NAR virus database (https://digitalworldbiology.com/blog/bio-databases-2020-viruses-and-covid-19). Summed contig reads per kilobase per million mapped reads values were calculated for each species based on the annotations. Oligonucleotide primers were then designed based on the mycovirus contigs. Reverse transcription (RT)-PCR was performed to determine whether the target regions could be amplified. Amplicons were purified using a gel extraction kit (TaKaRa, Dalian, China), ligated to the PMD18-T vector, and sent to the Shanghai Biotechnology Corporation for sequencing. Most of the sequences of the four dsRNA fragments of the mycovirus isolated from *Trichoderma* strain HB40111 were obtained. The missing 5′- and 3′-terminal end sequences were resolved using 5′- and 3′-rapid amplification of cDNA ends (RACE) cloning (52, 53). Using Vector NTI Advance 11.5.4, contiguous sequences for the single RNAs were assembled into four complete sequences (dsRNA1, dsRNA2, dsRNA3, and dsRNA4), yielding the complete viral genome. The PCR products were sent to TsingKe Biological Technology (Beijing, China) for sequencing.

### Phylogenetic analysis.

Putative open reading frames (ORFs) were predicted with the ORF Finder tool (https://www.bioinformatics.org/sms2/orf_Find) (54). The nucleotide mycovirus sequences were used as queries in BLASTN analysis (https://blast.ncbi.nlm.nih.gov/ BLAST.cgi) on the United States National Center for Biotechnology Information (NCBI) website (https://blast.ncbi.nlm.nih.gov/Blast.cgi) to identify similar mycoviruses for inclusion in the phylogenetic analysis. All sequences that were derived from fungal dsRNA viruses, had similar sizes to the genomic fragments observed on the agarose gel, and had a similarity higher than 40% were selected for further processing. Phylogenetic trees were constructed with the best models based on the amino acid on the single ORFs (ORF1, ORF2, ORF3, and ORF4) and a concatenated ORF1+ORF2+ORF3+ORF4 of the 12 mycovirus isolates from *Trichoderma* spp. alignment using the maximum likelihood (ML) method implemented in MEGA v10.0 (https://megasoftware.net/) (55) with 1000 bootstrap replicates. The best amino acid substitution models were LG+G+I+F (ORF1), LG+G+F (ORF2, ORF3, and a concatenated sequence), and WAG+G (ORF4) (Table S2).

### RNA and protein structure predictions.

Mycovirus terminal RNA sequence structures were predicted for each genomic fragment using RNA structure v6.4 ([56], http://rna.urmc.rochester.edu/RNAstructure. Html). The 3D structure of proteins putatively encoded by ORF1, ORF2, ORF3, and ORF4 were predicted using I-TASSER (https://zhanggroup.org) (57). The target proteins were annotated with COFACTOR (Roy, 2012a; Roy, 2012b), COACH (58), ConCavity (59), FINDSITE (60), Firestar (61), 3DLigandSite (62), and I-TASSER-MTD (63) implemented in the Zhang Lab Online Service (https://zhanggroup.org/services/) (44). The annotations included predicted protein functions, ligand-binding sites, and active site residues.

### Viral nomenclature.

The name of the novel mycovirus was assigned according to the International Code of Virus Classification and Nomenclature (ICVCN) assessed through the website of the International Committee on Taxonomy of Viruses (https://ictv.global/about/code) and their guidelines (https://ictv.global/faq/names).

### Data availability.

The nucleotide and amino acid sequences of ORF1, ORF2, ORF3, and ORF4 of the novel mycovirus isolated in this study have been submitted to GenBank under the accession numbers OM307406, OM307407, OM307408, and OM307409, respectively. The proteins encoded by ORF1, ORF2, ORF3, and ORF4 were also assigned the accession numbers WAA18813, WAA18814, WAA18815, and WAA18816, respectively.
