# Supplementary material for: An Outstandingly Rare Occurrence of Mycoviruses in Soil Strains of the Plant-Beneficial Fungi from the Genus Trichoderma and a Novel Polymycoviridae Isolate
Source: Microbiol Spectr. 2023 Apr 6;11(3):e05228-22. doi: 10.1128/spectrum.05228-22 (PMC10269472; doi:10.1128/spectrum.05228-22)

SUPPLEMENTARY TABLES

Table S1. The information on the *Trichoderma* strains collected from Xinjiang, Inner Mongolia, Jilin and Heilongjiang provinces of China

Strain Number	ITS-based identification	Region	Containing mycovirus
CTCCSJ-G-QT40002	*Harzianum* Clade	Xinjiang	
CTCCSJ-G-QT40003	*Harzianum* Clade	Xinjiang	
CTCCSJ-G-QT40004	*Harzianum* Clade	Xinjiang	
CTCCSJ-G-HB40005	Section *Trichoderma*	Xinjiang	
CTCCSJ-F-ZY40007	Section *Longibrachiatum*	Xinjiang	
CTCCSJ-F-ZY40008	Section *Longibrachiatum*	Xinjiang	
CTCCSJ-F-ZY40009	Section *Longibrachiatum*	Xinjiang	
CTCCSJ-G-JK40012	*Harzianum* Clade	Xinjiang	
CTCCSJ-G-HB40015	*Harzianum* Clade	Xinjiang	
CTCCSJ-G-HB40017	*Harzianum* Clade	Xinjiang	
CTCCSJ-G-HB40019	*Harzianum* Clade	Xinjiang	
CTCCSJ-F-ZY40020	Section *Longibrachiatum*	Xinjiang	
CTCCSJ-F-KY40021	Section *Longibrachiatum*	Xinjiang	
CTCCSJ-F-ZY40022	Section *Longibrachiatum*	Xinjiang	
CTCCSJ-F-ZY40023	Section *Longibrachiatum*	Xinjiang	
CTCCSJ-G-HB40030	*Harzianum* Clade	Inner Mongolia	
CTCCSJ-G-HB40031	*Harzianum* Clade	Inner Mongolia	
CTCCSJ-G-HB40032	Section *Longibrachiatum*	Inner Mongolia	
CTCCSJ-G-HB40038	Section *Longibrachiatum*	Inner Mongolia	
CTCCSJ-G-HB40040	Section *Longibrachiatum*	Inner Mongolia	
CTCCSJ-G-HB40046	Section *Longibrachiatum*	Inner Mongolia	
CTCCSJ-F-ZY40048	*Harzianum* Clade	Xinjiang	
CTCCSJ-G-HB40050	*Harzianum* Clade	Inner Mongolia	
CTCCSJ-F-KY40053	*Harzianum* Clade	Inner Mongolia	
CTCCSJ-G-HB40055	*Harzianum* Clade	Xinjiang	
CTCCSJ-G-HB40057	Section *Longibrachiatum*	Inner Mongolia	
CTCCSJ-G-HB40061	*Harzianum* Clade	Inner Mongolia	
CTCCSJ-G-HB40065	*Harzianum* Clade	Inner Mongolia	
CTCCSJ-G-JK40067	*Harzianum* Clade	Xinjiang	
CTCCSJ-G-HB40091	*Harzianum* Clade	Inner Mongolia	
CTCCSJ-G-HB40111	*T. barbatum**	Inner Mongolia	Trichoderma barbatum polymycovirus 1 - *Polymycoviridae*
CTCCSJ-G-QT40119	Section *Trichoderma*	Jilin	
CTCCSJ-F-ZY40121	Section *Trichoderma*	Jilin	

Strain Number	ITS-based identification	Region	Containing mycovirus
CTCCSJ-G-HB40137	*T. harzianum*	Xinjiang	Trichoderma harzianum bipartite mycovirus 1-unclassified mycovirus
CTCCSJ-G-HB40142	*Harzianum* Clade	Xinjiang	
CTCCSJ-G-QT40148	Section *Longibrachiatum*	Inner Mongolia	
CTCCSJ-G-QT40151	Section *Longibrachiatum*	Inner Mongolia	
CTCCSJ-F-KY40153	Section *Longibrachiatum*	Inner Mongolia	
CTCCSJ-G-QT40154	Section *Longibrachiatum*	Inner Mongolia	
CTCCSJ-F-KY40155	Section *Longibrachiatum*	Inner Mongolia	
CTCCSJ-G-HB40167	Section *Longibrachiatum*	Inner Mongolia	
CTCCSJ-G-QT40168	Section *Longibrachiatum*	Inner Mongolia	
CTCCSJ-G-HB40188	Section *Longibrachiatum*	Xinjiang	
CTCCSJ-G-HB40189	Section *Longibrachiatum*	Xinjiang	
CTCCSJ-G-HB40190	Section *Longibrachiatum*	Xinjiang	
CTCCSJ-G-QT40192	Section *Longibrachiatum*	Xinjiang	
CTCCSJ-G-QT40194	Section *Longibrachiatum*	Inner Mongolia	
CTCCSJ-G-HB40218	*T.* sp.	Inner Mongolia	
CTCCSJ-G-QT40222	Section *Longibrachiatum*	Inner Mongolia	
CTCCSJ-G-QT40223	Section *Longibrachiatum*	Inner Mongolia	
CTCCSJ-G-HB40226	Section *Longibrachiatum*	Xinjiang	
CTCCSJ-G-JK40241	Section *Longibrachiatum*	Xinjiang	
CTCCSJ-G-JK40242	Section *Longibrachiatum*	Xinjiang	
CTCCSJ-G-HB40244	*Harzianum* Clade	Inner Mongolia	
CTCCSJ-F-ZY40245	*Harzianum* Clade	Inner Mongolia	
CTCCSJ-F-ZY40246	*Harzianum* Clade	Xinjiang	
CTCCSJ-F-ZY40248	Section *Longibrachiatum*	Xinjiang	
CTCCSJ-G-QT40252	*Harzianum* Clade	Inner Mongolia	
CTCCSJ-G-JK40253	*Harzianum* Clade	Xinjiang	
CTCCSJ-G-HB40257	Section *Longibrachiatum*	Inner Mongolia	
CTCCSJ-G-HB40263	Section *Longibrachiatum*	Inner Mongolia	
CTCCSJ-F-ZY40265	Section *Longibrachiatum*	Xinjiang	
CTCCSJ-G-HB40270	Section *Longibrachiatum*	Xinjiang	
CTCCSJ-G-QT40271	Section *Longibrachiatum*	Inner Mongolia	
CTCCSJ-G-QT40274	Section *Longibrachiatum*	Inner Mongolia	
CTCCSJ-G-HB40275	Section *Longibrachiatum*	Inner Mongolia	
CTCCSJ-G-HB40276	Section *Longibrachiatum*	Xinjiang	
CTCCSJ-F-KY40280	Section *Longibrachiatum*	Inner Mongolia	
CTCCSJ-G-QT40283	Section *Longibrachiatum*	Inner Mongolia	
CTCCSJ-G-HB40284	*Harzianum* Clade	Inner Mongolia	
CTCCSJ-G-JK40288	*Harzianum* Clade	Xinjiang	

Strain Number	ITS-based identification	Region	Containing mycovirus
CTCCSJ-G-QT40290	*Harzianum* Clade	Inner Mongolia	
CTCCSJ-G-JK40292	*Harzianum* Clade	Xinjiang	
CTCCSJ-F-ZY40300	*Harzianum* Clade	Inner Mongolia	
CTCCSJ-G-DK40302	Section *Longibrachiatum*	Inner Mongolia	
CTCCSJ-G-QT40303	Section *Trichoderma*	Xinjiang	
CTCCSJ-G-QT40304	Section *Trichoderma*	Inner Mongolia	
CTCCSJ-G-HB40311	Section *Trichoderma*	Heilongjiang	
CTCCSJ-G-QT40314	Section *Trichoderma*	Inner Mongolia	
CTCCSJ-G-QT40315	Section *Trichoderma*	Inner Mongolia	
CTCCSJ-G-HB40322	Section *Trichoderma*	Xinjiang	
CTCCSJ-G-QT40323	Section *Trichoderma*	Jilin	
CTCCSJ-G-QT40324	Section *Trichoderma*	Inner Mongolia	
CTCCSJ-G-HB40325	Section *Trichoderma*	Inner Mongolia	
CTCCSJ-G-HB40327	Section *Trichoderma*	Inner Mongolia	
CTCCSJ-G-HB40340	*Harzianum* Clade	Inner Mongolia	
CTCCSJ-F-ZY40342	*Harzianum* Clade	Xinjiang	
CTCCSJ-F-ZY40354	*Harzianum* Clade	Xinjiang	
CTCCSJ-G-QT40356	*Harzianum* Clade	Inner Mongolia	
CTCCSJ-F-ZY40358	Section *Longibrachiatum*	Jilin	
CTCCSJ-G-HB40372	Section *Longibrachiatum*	Xinjiang	
CTCCSJ-G-HB40373	Section *Longibrachiatum*	Inner Mongolia	
CTCCSJ-G-JK40376	*Harzianum* Clade	Xinjiang	
CTCCSJ-G-QT40408	Section *Longibrachiatum*	Inner Mongolia	
CTCCSJ-G-HB40421	Section *Trichoderma*	Xinjiang	
CTCCSJ-G-HB40423	Section *Trichoderma*	Xinjiang	
CTCCSJ-G-HB40425	*T.* sp.	Xinjiang	
CTCCSJ-G-HB40429	Section *Longibrachiatum*	Xinjiang	
CTCCSJ-G-HB40430	Section *Trichoderma*	Xinjiang	
CTCCSJ-F-ZYB40431	Section *Trichoderma*	Xinjiang	
CTCCSJ-G-HB40433	*Harzianum* Clade	Xinjiang	
CTCCSJ-G-QT40435	*Harzianum* Clade	Xinjiang	
CTCCSJ-G-HB40436	Section *Trichoderma*	Xinjiang	
CTCCSJ-F-ZY40439	Section *Trichoderma*	Xinjiang	
CTCCSJ-G-HB40440	Section *Trichoderma*	Xinjiang	
CTCCSJ-G-HB40441	*Harzianum* Clade	Xinjiang	
CTCCSJ-G-QT40442	*Harzianum* Clade	Xinjiang	
CTCCSJ-G-HB40444	*Harzianum* Clade	Xinjiang	Not identified, unpublished
CTCCSJ-G-QT40447	*Harzianum* Clade	Xinjiang	

Strain Number	ITS-based identification	Region	Containing mycovirus
CTCCSJ-G-HB40448	Section *Trichoderma*	Xinjiang	
CTCCSJ-F-ZY40451	Section *Trichoderma*	Xinjiang	
CTCCSJ-F-ZY40453	Section *Trichoderma*	Xinjiang	
CTCCSJ-G-HB40455	*Harzianum* Clade	Xinjiang	
CTCCSJ-G-HB40456	Section *Trichoderma*	Xinjiang	
CTCCSJ-G-HB40461	*Harzianum* Clade	Inner Mongolia	
CTCCSJ-G-HB40462	*Harzianum* Clade	Inner Mongolia	
CTCCSJ-G-QT40476	Section *Trichoderma*	Inner Mongolia	
CTCCSJ-G-HB40478	*Harzianum* Clade	Inner Mongolia	
CTCCSJ-G-HB40481	*Harzianum* Clade	Inner Mongolia	
CTCCSJ-G-HB40482	*Harzianum* Clade	Inner Mongolia	
CTCCSJ-G-HB40483	*Harzianum* Clade	Inner Mongolia	
CTCCSJ-G-HB40484	*T.* cf. *rossicum*	Inner Mongolia	
CTCCSJ-G-HB40485	Section *Trichoderma*	Inner Mongolia	
CTCCSJ-G-QT40486	Section *Trichoderma*	Inner Mongolia	
CTCCSJ-G-HB40487	*Harzianum* Clade	Inner Mongolia	
CTCCSJ-G-HB40490	*Harzianum* Clade	Inner Mongolia	
CTCCSJ-G-HB40495	Section *Trichoderma*	Inner Mongolia	
CTCCSJ-G-HB40496	Section *Longibrachiatum*	Inner Mongolia	
CTCCSJ-F-ZY40503	Section *Longibrachiatum*	Inner Mongolia	
CTCCSJ-G-JK40193	Section *Longibrachiatum*	Xinjiang	
XZ N236-1	Section *Trichoderma*	Inner Mongolia	
PDA N239-3	Section *Trichoderma*	Inner Mongolia	
CTCCSJ-G-QT40513	Section *Trichoderma*	Inner Mongolia	
CTCCSJ-G-HB40519	*Harzianum* Clade	Inner Mongolia	
CTCCSJ-G-HB40525	*Harzianum* Clade	Inner Mongolia	Trichoderma harzianum mycovirus 1-unclassified mycovirus
CTCCSJ-G-HB40547	*Harzianum* Clade	Xinjiang	
CTCCSJ-G-HB40550	Section *Trichoderma*	Xinjiang	
CTCCSJ-G-HB40551	*Harzianum* Clade	Xinjiang	
CTCCSJ-G-HB40565	*Harzianum* Clade	Xinjiang	
CTCCSJ-G-HB40582	*Harzianum* Clade	Xinjiang	
CTCCSJ-G-HB40609	*Harzianum* Clade	Xinjiang	
CTCCSJ-G-HB40613	*Harzianum* Clade	Xinjiang	
CTCCSJ-G-HB40614	*Harzianum* Clade	Xinjiang	
CTCCSJ-G-HB40615	*Harzianum* Clade	Xinjiang	
CTCCSJ-G-HB40616	*Harzianum* Clade	Xinjiang	
CTCCSJ-G-HB40618	*Harzianum* Clade	Xinjiang	
CTCCSJ-G-QT40623	*Harzianum* Clade	Xinjiang	

Strain Number	ITS-based identification	Region	Containing mycovirus
CTCCSJ-G-QT40624	Lone lineage	Xinjiang	
CTCCSJ-F-KZ40643	*Harzianum* Clade	Xinjiang	
CTCCSJ-G-JK40647	Section *Trichoderma*	Xinjiang	
CTCCSJ-G-HB40732	*Harzianum* Clade	Xinjiang	
CTCCSJ-F-KZ40769	Section *Trichoderma*	Xinjiang	
CTCCSJ-G-HB40733	*Harzianum* Clade	Xinjiang	
CTCCSJ-F-KZ40809	*Harzianum* Clade	Xinjiang	
CTCCSJ-G-QT40916	*Harzianum* Clade	Inner Mongolia	
CTCCSJ-G-HB40925	*Harzianum* Clade	Inner Mongolia	
CTCCSJ-G-HB40930	*Harzianum* Clade	Inner Mongolia	
CTCCSJ-G-HB40941	*Harzianum* Clade	Inner Mongolia	
CTCCSJ-G-JK40972	*Harzianum* Clade	Inner Mongolia	
CTCCSJ-G-JK40974	*Harzianum* Clade	Inner Mongolia	
CTCCSJ-G-HB40989	*Harzianum* Clade	Inner Mongolia	
CTCCSJ-G-QT40994	Section *Trichoderma*	Heilongjiang	
CTCCSJ-G-HB41044	*Harzianum* Clade	Inner Mongolia	

* precise identification achived based on the analysis of *tef1* and *rpb2* DNA barcoding markers as pecified in Cai & Druzhinina, 2021. The details are presented in the main ms text.

Table S2. Parameters of the best models used for the phylogenetic analysis of each locus.

ORF	Best model	Bayesian Information Criterion (BIC)	Gamma (α value)	Frequency of invariant sites (I)	Rate of amino acid substitutions (R)
ORF1	LG+G+I+F	55313.41831	1.209802903	0.001090594	0.075277057
ORF2	LG+G+F	24718.36565	1.587579446	0.036937223	0.073398859
ORF3	WAG+G	7680.337321	1.734298803	n/a	0.043972
ORF1+ORF2 +ORF3	LG+G+F	56509.98844	1.22091035	n/a	0.077131838

Table S3. Metadata for the sequences used in the ORF1 phylogenetic analysis.

**Accession Number**	**Mycovirus species**	**Host species**
QAU09249.1	Magnaporthe oryzae polymycovirus 1	*Magnaporthe oryzae*
CUS18599.1	Beauveria bassiana polymycovirus 2	*Beauveria bassiana*
QRF54813.1	Beauveria bassiana polymycovirus 4	*B. bassiana*
CAD7829823.1	Beauveria bassiana polymycovirus 3	*B. bassiana*
BCH36613.1	Aspergillus fumigatus polymycovirus 1	*Aspergillus fumigatus*
YP_009551547.1	Aspergillus fumigatus polymycovirus 1	*A. fumigatus*
YP_009052470.1	Cladosporium cladosporioides virus 1	*Cladosporium cladosporioides*
ASV63092.1	Colletotrichum camelliae filamentous virus 1	*Colletotrichum camelliae*
AYP71801.1	Penicillium brevicompactum tetramycovirus 1	*Penicillium brevicompactum*
QDH44656.1	Fusarium redolens polymycovirus 1	*Fusarium redolens*
BCJ03666.1	Penicillium janthinellum polymycovirus 1	*Penicillium janthinellum*
QHG11074.1	Plasmopara viticola lesion associated polymycovirus 5	*Plasmopara viticola*
YP_009551548.1	Penicillium digitatum polymycoviruses 1	*Penicillium digitatum*
AWY10945.1	Sclerotinia sclerotiorum tetramycovirus-1	*Sclerotinia sclerotiorum*
YP_009352879.1	Beauveria bassiana polymycovirus 1	*B. bassiana*
AJP08049.1	Alternaria tenuissima virus	*Alternaria tenuissima*
BCM78286.1	Hadaka virus 1	*Hadaka*
BBU94038.1	Hadaka virus 1	*Hadaka*
QHG11073.1	Plasmopara viticola lesion associated polymycovirus 4	*P. viticola*
QED43024.1	Uromyces virus B	*Uromyces*
AVV48358.1	Pepper cryptic virus 1	*Pepper cryptic*
QEO60284.1	Pepper cryptic virus 1	*Pepper cryptic*
AVL84362.1	Pepper cryptic virus 1	*Pepper cryptic*

Table S4. Metadata for the sequences used in the ORF2 phylogenetic analysis.

**Accession Number**	**Mycovirus species**	**Host species**
QAU09251.1	Magnaporthe oryzae polymycovirus 1	*Magnaporthe oryzae*
QRF54815.1	Beauveria bassiana polymycovirus 4	*Beauveria bassiana*
CAD7829825.1	Beauveria bassiana polymycovirus 3	*B. bassiana*
YP_009052472.1	Cladosporium cladosporioides virus 1	*Cladosporium cladosporioides*
ASV63094.1	Colletotrichum camelliae filamentous virus 1	*Colletotrichum camelliae*
AWY10947.1	Sclerotinia sclerotiorum tetramycovirus-1	*Sclerotinia sclerotiorum*
QHG11068.1	Plasmopara viticola lesion associated polymycovirus 1	*Plasmopara viticola*
QDB74987.1	Phaeoacremonium minimum tetramycovirus 1	*Phaeoacremonium minimum*
AYP71807.1	Aspergillus spelaeus tetramycovirus 1	*Aspergillus spelaeus*
QED42895.1	Phakopsora virus B	*Phakopsora*
BCM78288.1	Hadaka virus 1	*Hadaka*
BBU94040.1	Hadaka virus 1	*Hadaka*

Table S5. Metadata for the sequences used in the ORF3 phylogenetic analysis.

**Accession Number**	**Mycovirus species**	**Host species**
QAU09252.1	Magnaporthe oryzae polymycovirus 1	*Magnaporthe oryzae*
QRF54816.1	Beauveria bassiana polymycovirus 4	*Beauveria bassiana*
CAD7829826.1	Beauveria bassiana polymycovirus 3	*B. bassiana*
BCH36614.1	Aspergillus fumigatus polymycovirus 1	*Aspergillus fumigatus*
YP_009551545.1	Aspergillus fumigatus polymycovirus 1	*A. fumigatus*
YP_009052473.1	Cladosporium cladosporioides virus 1	*Cladosporium cladosporioides*
ASV63095.1	Colletotrichum camelliae filamentous virus 1	*Colletotrichum camelliae*
AWY10946.1	Sclerotinia sclerotiorum tetramycovirus-1	*Sclerotinia sclerotiorum*
QDB74988.1	Phaeoacremonium minimum tetramycovirus 1	*Phaeoacremonium minimum*
CDP74621.1	Aspergillus fumigatus tetramycovirus-1	*A. fumigatus*
AYP71806.1	Aspergillus spelaeus tetramycovirus 1	*Aspergillus spelaeus*
QRF54818.1	Beauveria bassiana polymycovirus 4	*B. bassiana*

SUPPLEMENTARY FIGURES

Figure S1.

ITS-based cladogram showing the diversity of 160 *Trichoderma* spp. strains isolated in this study. The mycovirus-positive strain is shown in bold. The detailed identification of mycovirus-free strains is presented elsewhere.

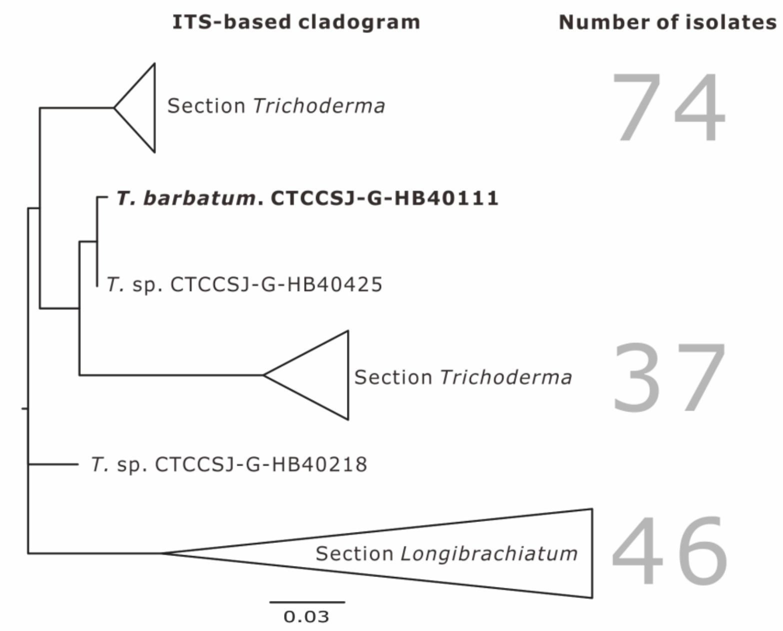

Figure S2.
Structure of the 5’ UTR of dsRNA1 analyzed using I-TASSER.

Minimum free energy of the structure= -5.80 kcal/mol

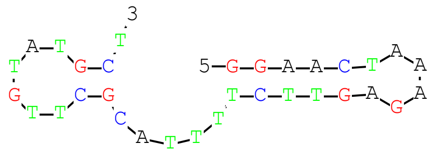

Figure S3.
Structure of the 3’ UTR of dsRNA1 analyzed using I-TASSER.
Minimum free energy of the structure= -44.00 kcal/mol

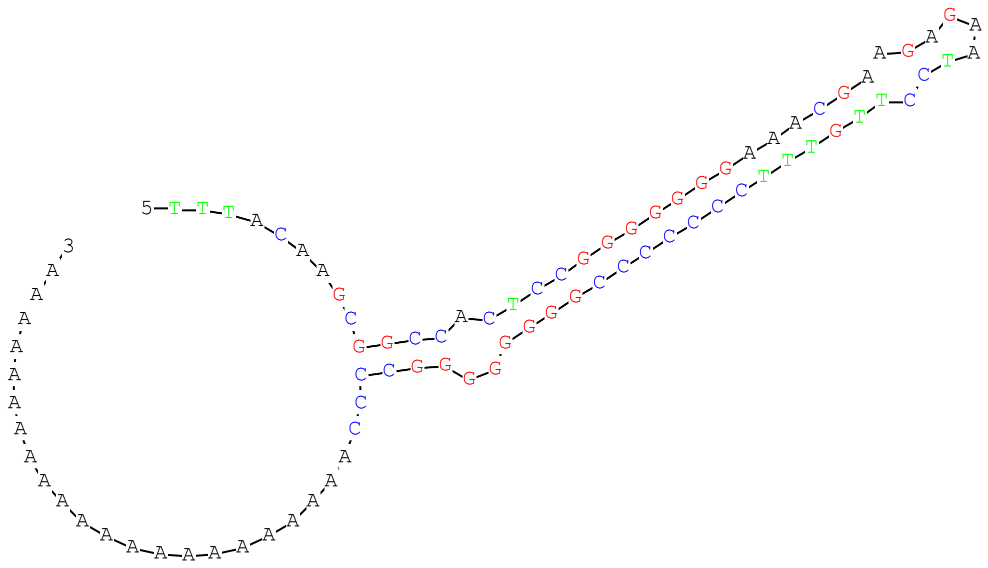

Figure S4.
Structure of the 5’ UTR of dsRNA2 analyzed using by I-TASSER.

```
Minimum free energy of the structure= -15.60 kcal/mol
```

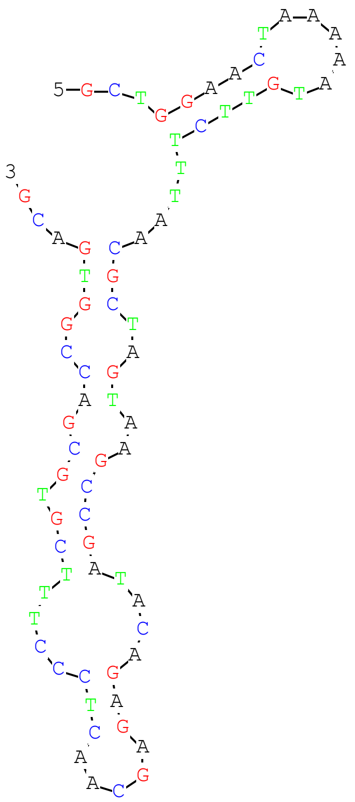

Figure S5.
Structure of the 3’ UTR of dsRNA2 analyzed using I-TASSER.

```
Minimum free energy of the structure= -15.50 kcal/mol
```

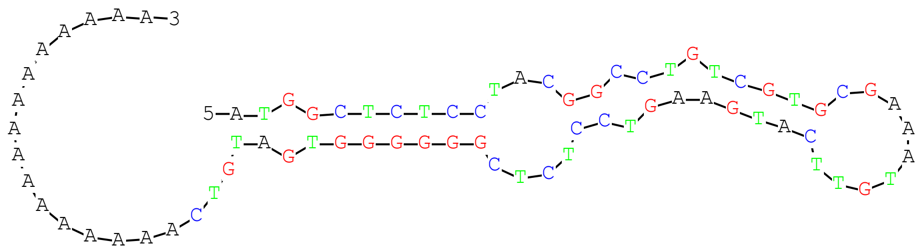

Figure S6.
Structure of the 5' UTR of dsRNA3 analyzed using I-TASSER.

Minimum free energy of the structure= -1.20 kcal/mol

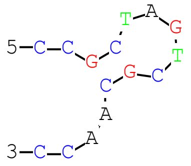

Figure S7.
Structure of the 3' UTR of dsRNA3 analyzed using I-TASSER.

Minimum free energy of the structure= -5.90 kcal/mol

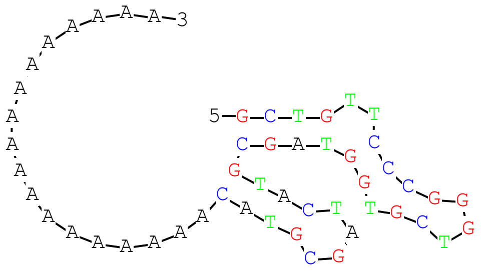

Figure S8.
Structure of the 5' UTR of dsRNA4 analyzed using I-TASSER.

Minimum free energy of the structure= -75.50 kcal/mol

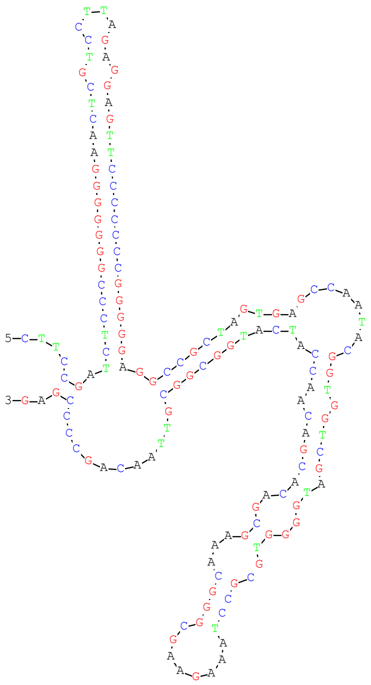

Figure S9.

Structure of the 3’ UTR of dsRNA4 analyzed using I-TASSER.

```
Minimum free energy of the structure= -24.60 kcal/mol
```

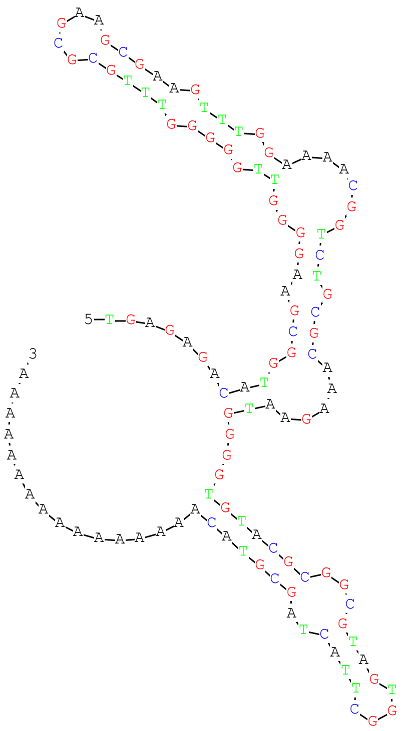

Figure S10.

Structure of the protein product of ORF1 using I-TASSER (A putative RNA-dependent RNA polymerase. C-score = -2.68; estimated TM-score = 0.41 ± 0.14; estimated root mean square deviation (RMSD) = 15.1 ± 3.5 Å.)

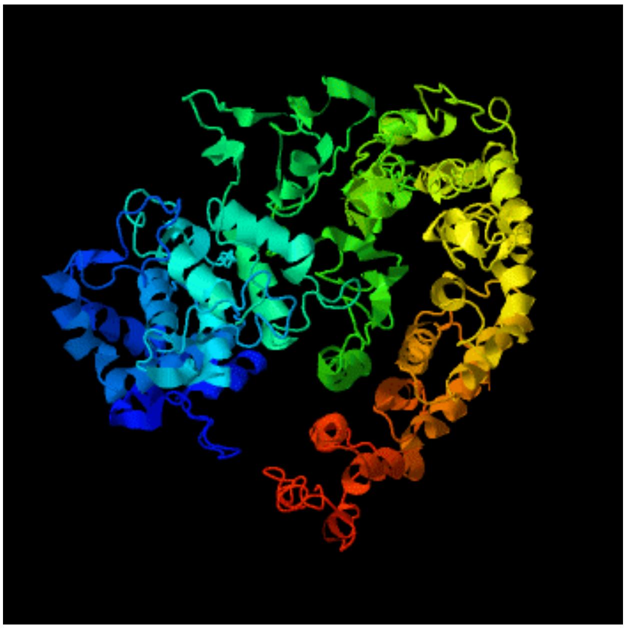

Figure S11.

Structure of the protein product of ORF2 using I-TASSER (A hypothetical protein. C-score = -3.01; estimated TM-score = 0.37 ± 0.13; estimated root mean square deviation (RMSD) = 15.6 ± 3.3 Å)

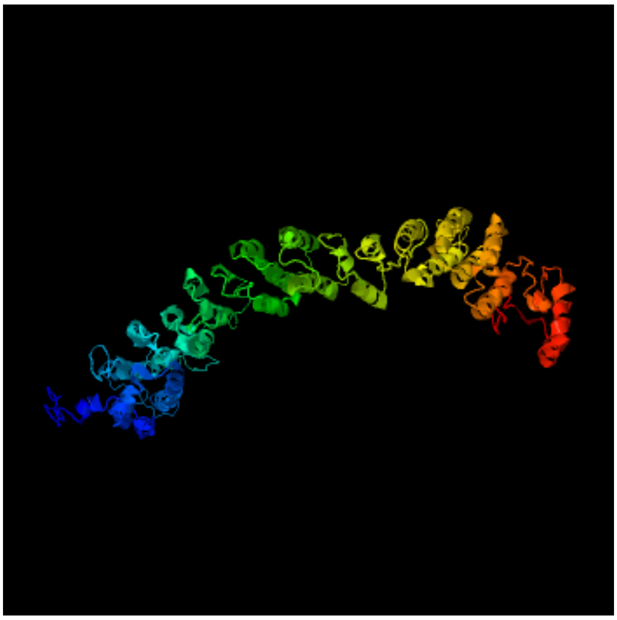

Figure S12.
Structure of the protein product of ORF3 using I-TASSER (a hypothetical protein. C-score = -1.98; estimated TM-score = 0.48 ± 0.15; estimated root mean square deviation (RMSD) = 12.6 ± 4.3 Å)

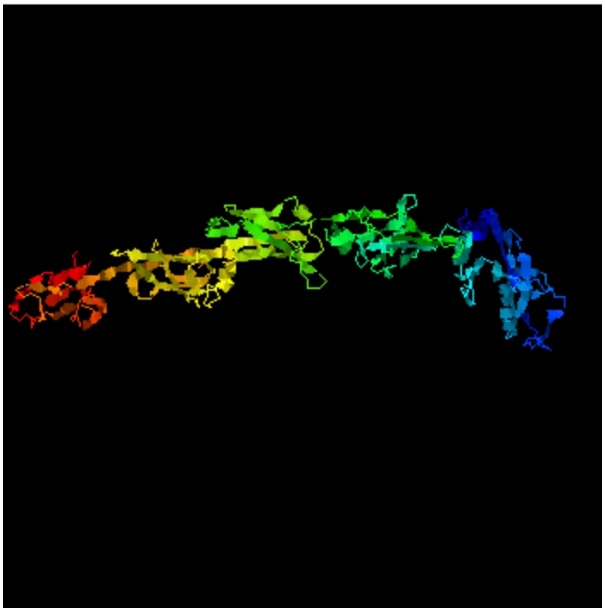

Figure S13.
Structure of the protein product of ORF4 using I-TASSER (A hypothetical protein. C-score = -4.80; estimated TM-score = 0.22 ± 0.06; estimated root mean square deviation (RMSD) = 18.0 ± 2.4 Å)

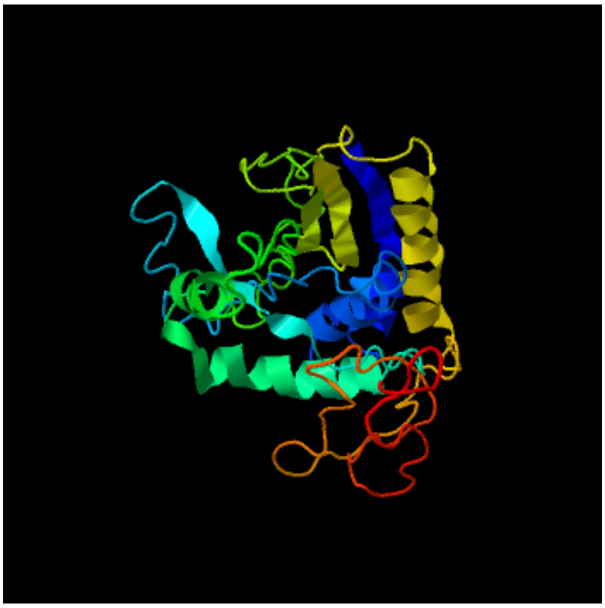

Supplement: Supplemental file 1 — Supplemental material. Download spectrum.05228-22-s0001.pdf, PDF file, 0.5 MB [file spectrum.05228-22-s0001.pdf]
